# P27^Kip1^, regulated by glycogen synthase kinase-3β, results in HMBA-induced differentiation of human gastric cancer cells

**DOI:** 10.1186/1471-2407-11-109

**Published:** 2011-03-27

**Authors:** Min Wei, Zhiwei Wang, Hongliang Yao, Zhongyin Yang, Qing Zhang, Bingya Liu, Yingyan Yu, Liping Su, Zhenggang Zhu, Qinlong Gu

**Affiliations:** 1 Key Laboratory of Shanghai Gastric Neoplasms, Department of Surgery, Shanghai Institute of Digestive Surgery, Ruijin Hospital, Shanghai Jiao Tong University School of Medicine, Shanghai, 200025, China; 2 School of Science, Nanjing University of Science and Technology, Nanjing, 210094, China

**Keywords:** HMBA, gastric cancer, GSK-3β

## Abstract

**Background:**

Gastric cancer is the second most common cause of global cancer-related mortality. Although dedifferentiation predicts poor prognosis in gastric cancer, the molecular mechanism underlying dedifferentiation, which could provide fundamental insights into tumor development and progression, has yet to be elucidated. Furthermore, the molecular mechanism underlying the effects of hexamethylene bisacetamide (HMBA), a recently discovered differentiation inducer, requires investigation and there are no reported studies concerning the effect of HMBA on gastric cancer.

**Methods:**

Based on the results of FACS analysis, the levels of proteins involved in the cell cycle or apoptosis were determined using western blotting after single treatments and sequential combinations of HMBA and LiCl. GSK-3β and proton pump were investigated by western blotting after up-regulating Akt expression by Ad-Akt infection. To investigate the effects of HMBA on protein localization and the activities of GSK-3β, CDK2 and CDK4, kinase assays, immunoprecipitation and western blotting were performed. In addition, northern blotting and RNase protection assays were carried out to determine the functional concentration of HMBA.

**Results:**

HMBA increased p27Kip1 expression and induced cell cycle arrest associated with gastric epithelial cell differentiation. In addition, treating gastric-derived cells with HMBA induced G0/G1 arrest and up-regulation of the proton pump, a marker of gastric cancer differentiation. Moreover, treatment with HMBA increased the expression and activity of GSK-3β in the nucleus but not the cytosol. HMBA decreased CDK2 activity and induced p27Kip1 expression, which could be rescued by inhibition of GSK-3β. Furthermore, HMBA increased p27Kip1 binding to CDK2, and this was abolished by GSK-3β inhibition.

**Conclusions:**

The results presented herein suggest that GSK-3β functions by regulating p27Kip1 assembly with CDK2, thereby playing a critical role in G0/G1 arrest associated with HMBA-induced gastric epithelial cell differentiation.

## Background

Gastric cancer is one of the most common cancers in the world and often develops resistance to chemotherapy and radiation treatments. Therefore, combination therapy has been proposed to tackle the disease better and to reduce the probability of developing resistance [1]. Hexamethylene bisacetamide (HMBA), a hybrid polar compound (HPC) originally developed as a differentiation-inducing agent [2-6], causes gastric cell re-differentiation [7-9].

In the stomach, stem cells in the proliferative cell zone of the isthmus region of the gastric glands differentiate and give rise to various cell types [10,11]. Once the first tumorigenic event takes place, further tumor progression depends on the nature of the initiating event and the developmental stage of the cell that sustained it and additional mutations that could occur. Constant proliferation is a vital feature of stem cells, and in gastrointestinal tissues mutations are likely to result in expansion of altered stem cells, increasing the probability of additional mutations and tumor progression [12]. Therefore, targeting gastric cancer stem cells is likely to be the most effective way of treating gastric cancer. Approximately 50% of the western population develops metaplasia, a key step in cancer development [13], drawing attention to pathways that control proliferation and thereby cell differentiation. Among these, the TGF-b, Myb, Wnt and Hedgehog pathways are of particular relevance, featuring prominently in cell-fate specification and pattern formation during embryogenesis and adult tissue renewal. The elucidation of complex tumor suppressor and accelerator signaling pathways, which effect differentiation modulation of transitional/progenitor cells, will be pivotal for optimization of therapeutics to treat gastric cancer.

In order for immature gastric cells to differentiate, they require to stay in the G1 phase of the cell cycle for a certain time period. The mammalian cell cycle is regulated by sequential activation and inactivation of a highly conserved family of cyclin dependent kinases (CDKs); progression through early to mid-G1 is dependent on CDK4 and possibly CDK6, while progression through late G1 and the S phase requires activation of CDK2. The activities of CDKs can be inhibited by the binding of CDK inhibitory proteins including the Cip/Kip family (p21^Waf1^, p27^Kip1 ^and p57^Kip2^) and INK4 family (p15Ink4b, p16Ink4a, p18Ink4c and p19Ink4d). P27^Kip1 ^is regulated post-transcriptionally by proteolytic degradation. CDK2 binds to p27^Kip1 ^and phosphorylates it on threonine 187 [14], and HMBA-induced gastric cell differentiation is associated with the up-regulation of p27^Kip1 ^[15,16] and G0/G1 arrest. However, there are few detailed studies concerning the molecular mechanism of HMBA and there have been no reported studies investigating the effect of HMBA on gastric cancer.

As a downstream target of the phosphatidylinositol-3 kinase/Akt (PI3-kinase/Akt) pathway, GSK-3β regulates cell proliferation and differentiation [17-20]. Accumulating evidence indicates that hypoactive GSK3β signaling, which functions in G1 to receive input from several signaling and developmental pathways, occurs in association with diverse human cancers [21,22]. GSK-3β has been implicated in multiple biological processes because it phosphorylates a broad range of substrates including several differentiation checkpoints including c-Myc, snail and PI3K [23]. Previously, inhibition of the PI3-kinase pathway was shown to enhance HMBA-mediated gastric cell differentiation [8]. In this study, the role of GSK-3β during gastric cell differentiation was investigated using the human gastric cancer cell line SGC7901, which displays a multipotent phenotype and represents a well-characterized model of gastric differentiation. The results suggest a contributory role for GSK-3β in the p27^Kip1 ^pathway during gastric cell differentiation induced by HMBA.

## Methods

### Cell culture

The gastric cancer cell line SGC7901 was obtained from the cell bank in the Chinese Academy of Sciences and cultured as previously described [24]. SGC7901 cells were infected with an adenovirus encoding the activated form of Akt (Ad-Akt) or the adenoviral control vector encoding β-galactosidase (β-gal) at a multiplicity of infection (MOI) of 10 pfu/cell. After infection with vectors for one hour, followed by replacement of medium and incubation for a further 24 h, cells were treated in the presence or absence of HMBA, and protein and RNA were extracted for western and Northern blotting, respectively.

### Materials

HMBA, TSA, SB-415286 and LiCl were purchased from Sigma Chemical Company, USA. Adenovirus vectors encoding β-gal and the myristoylated active form of Akt (Ad-Akt) were purchased from Cell BioLabs, USA. The vector encoding the catalytically active mutant of GSK3β (HA-GSK-3βCA) was purchased from Addgene. Non-targeting control siRNA and the SMARTpool for targeting GSK-3β were purchased from Dharmacon, USA. All probes were labeled with a Biotin Random Prime DNA Labeling Kit (Pierce).

### Antibodies

Rabbit anti-Akt, anti-phospho-Akt (Ser473) and antiphospho-GSK-3β (Ser9) were purchased from Cell Signaling, USA. Mouse anti-GSK-3β, mouse anti-p27^Kip1^, mouse anti-Top IIb and mouse anti-p21 ^Waf1 ^were purchased from BD Biosciences, USA. Mouse anti-phospho-GSK-3 (Tyr278/Tyr216) was obtained from Upstate, USA. Rabbit anti-cleaved PARP antibody was purchased from Abcam, USA. Anti-proton pump was bought from MBL International (USA). Polyclonal anti-CDK2, anti-CDK4, anti-α-tubulin and anti-caspase-3 were obtained from Santa Cruz Biotechnology (USA).

### Sub-cellular protein extraction and western blotting

Nuclear and cytosolic fractions were extracted using a NE-PER Nuclear and Cytoplasmic Extraction Reagents kit (Pierce, USA). Cytosolic protein (80 mg) or nuclear protein (20 mg) was resolved on a 10% polyacrylamide gel and transferred to PVDF membranes as previously described [25]. Filters were incubated for one hour at room temperature in blotting solution. Membranes were incubated overnight at 4°C with primary antibodies followed by blotting with a horseradish peroxidase-conjugated secondary antibody for one hour, and visualized using an ECL detection system.

### Northern blotting and RNase protection assays (RPA)

Total RNA was prepared using TRIzol reagent (Invitrogen). Samples were run on 1.2% agarose/formaldehyde gels and transferred to supported nitrocellulose. Membranes were hybridized with a biotin labeled gastric proton pump cDNA probe. After hybridization with the GAPDH probe, a loading control, membranes were washed and signals were detected using an ECL detection system. RNase protection experiments were performed using the RPA-III kit from Ambion and the RiboQuant MultiProbe RNase Protection Assay System was utilized to detect multiple specific mRNAs. ^32^P-labeled antisense RNA probes were prepared using the Human Apoptosis hCC-2 and hCYC-1 Template Sets and hybridizations were performed according to the manufacturer's protocol.

### Cell cycle analysis

Gastric cancer cells were harvested using trypsin. Cells were collected, washed twice with ice-cold PBS and fixed in ice-cold 70% ethanol. After being washed twice with ice-cold PBS, resuspended in PBS containing 100 U/ml RNase A and incubated at 37°C for 30 min, cells were stained with PI (20 mg/ml) and analyzed using FACScan (Becton Dickinson, San Jose, CA, USA), as previously described [25].

### *In vitro *kinase assays

The activities of CDK2, CDK4 and GSK-3β were measured as previously described [26,27]. Briefly, CDK2, CDK4 or GSK-3β was immunoprecipitated from cytosolic (100 mg of protein) or nuclear (25 mg of protein) extracts. Kinase activity was measured by incubating immunoprecipitated CDK2, CDK4 or GSK-3β in 40 ml of kinase buffer with 4 mg recombinant Snail protein (to measure GSK-3β-associated kinase activity), 5 mg of histone H1 (to measure CDK2-associated kinase activity) or retinoblastoma protein (to measure CDK4-associated kinase activity) at 30°C for 30 min. The samples were processed as described in previous reports [28].

## Results

### Inhibition of GSK-3β attenuates HMBA-induced cell cycle arrest and SGC7901 cell differentiation

**Figure 1:**
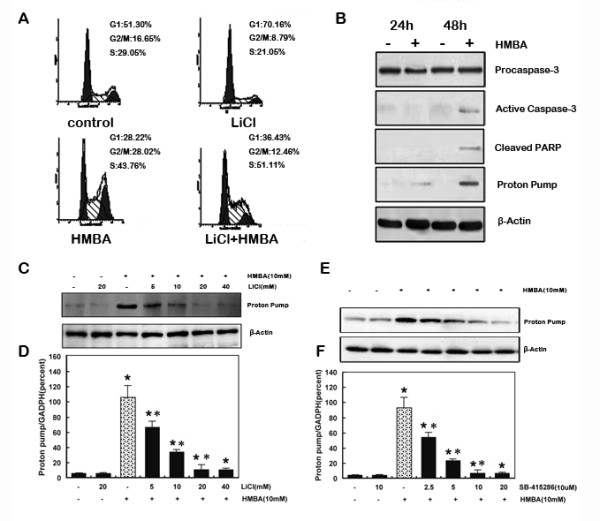
**Inhibition of GSK-3β attenuates HMBA-induced cell cycle arrest and SGC7901 cell differentiation**. **A**, SGC7901 cells were pre-treated with or without 10 mM LiCl for 30 min followed by combination treatment with 10 mM HMBA for 24 h, followed with quantifying of DNA content by flow cytometry. **B**, SGC7901 cells were treated with HMBA (10 mM) for 24 h or 48 h and were prepared for western blotting analysis. **C&E**, SGC7901 cells were pre-treated with or without 10 mM LiCl or 10 μMSB-415286 for one hour followed by combination treatment with 10 mMHMBA for 24 h. proton pump expression status was determined via western blotting analysis. **D&F**, total RNA was extracted from cells and Q-RT-PCR analysis for proton pump mRNA expression was performed. (Data represent mean ± SD; * = p < 0.05 vs. control; * = p < 0.05 vs. HMBA alone.).

SGC7901 cells accumulated at the G0/G1 cell cycle checkpoint and differentiated into an enterocyte-like phenotype after treatment with HMBA [29]. GSK-3β contributes to the inhibition of cell cycle progression in differentiating cells [20,30]. Therefore, whether GSK-3β plays a role in HMBA-induced SGC7901 cell cycle inhibition was investigated. As demonstrated in Figure 1a, treatment with HMBA induced cells to accumulate at the G0/G1 cell cycle checkpoint. Treatment with lithium chloride (LiCl), which inhibits GSK-3β in a Mg^2+ ^competitive manner [31], increased the proportion of cells in the S phase. Treatment with a combination of LiCl and HMBA reversed HMBA-mediated G1 cell arrest. Similar results were obtained after treatment with SB-415286, a potent inhibitor of GSK-3β [32] (Additional file 1). These results suggest that GSK-3β could play a role in HMBA-induced G1 arrest. To determine whether HMBA resulted in cell death during the 24 h treatment period, protein was extracted to assess whether there was increased PARP cleavage and/or active caspase-3. As demonstrated in Figure 1b, there was no increase in PARP cleavage and active caspase-3 until 48 h after HMBA treatment. An important early event in the terminal differentiation of cells is their withdrawal from the cell cycle [6]. Since GSK-3β is documented to play a role in cell cycle arrest [33], it was postulated that inhibition of GSK-3β could inhibit differentiation. Therefore the effects of GSK-3β inhibitors on the induction of HMBA-mediated gastric proton pump expression, a marker of gastric differentiation, were examined. SGC7901 cells were pre-treated with LiCl (Figure 1c and 1d) or SB-415286 (Figure 1e and 1f) at various concentrations for one hour, and then treated with HMBA for 24 h. LiCl inhibited HMBA-induced gastric proton pump expression in a dose-dependent manner. Consistent with these results, SB-415286 blocked gastric proton pump protein and mRNA expression, which was induced by HMBA. Taken together, these results indicate that GSK-3β plays an important role in HMBA-mediated gastric cell differentiation.

### Akt regulates gastric differentiation induced by HMBA

**Figure 2:**
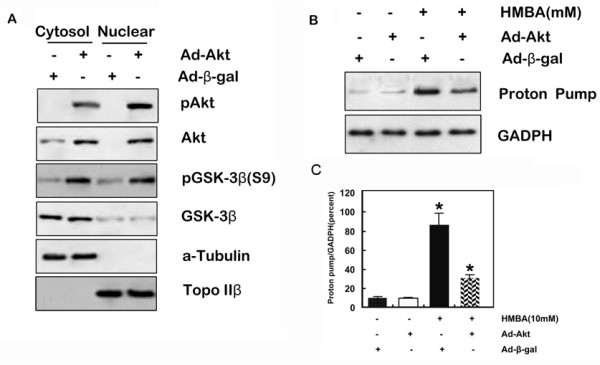
**Akt regulates gastric differentiation induced by HMBA**. **A**, Cytosol and nuclear proteins were extracted from cells treated as indicated and resolved on SDS-PAGE and blotted with anti-phospho-Akt, -Akt, phospho-GSK-3β, and GSK-3β, using anti-α-tubulin and Topo IIβ as control of the cytosolic and nuclear fractions respectively. **B**, proton pump expression was measured using western blotting followed with abundance quantification. (Data represent mean ± SD; * = p < 0.05 vs. control; † = p < 0.05 vs. HMBA alone.). **C**, Total RNA (40 μg) was fractionated, transferred to nitrocellulose membranes and probed with a labeled proton pump cDNA; blots were stripped and reprobed with GAPDH.

GSK-3β is inactivated when it is phosphorylated downstream of Akt [34]. Therefore, it would be predicted that activation of Akt by PI3-kinase would be associated with inhibition of GSK-3β and, subsequently, inhibition of gastric cell differentiation. To test this hypothesis, SGC7901 cells were infected with Ad-Akt or a control vector. Infection with Ad-Akt increased expression of phosphorylated Akt, Akt and phosphorylated GSK-3β protein (Figure 2a), consistent with previous results demonstrating that GSK-3β acts as a substrate of Akt. As demonstrated in Figure 2b, infection of SGC7901 cells with the Ad-Akt adenoviral vector alone had no effect on gastric proton pump and mRNA expression. However, infection with the Ad-Akt vector resulted in inhibition of gastric proton pump mRNA expression induced by HMBA compared with HMBA and infection of the control (β-gal) adenovirus, suggesting that signaling through the PI3-kinase/Akt pathway regulates gastric cell differentiation induced by HMBA treatment.

### Treatment with HMBA increased the expression and activity of GSK-3β in the nucleus

**Figure 3:**
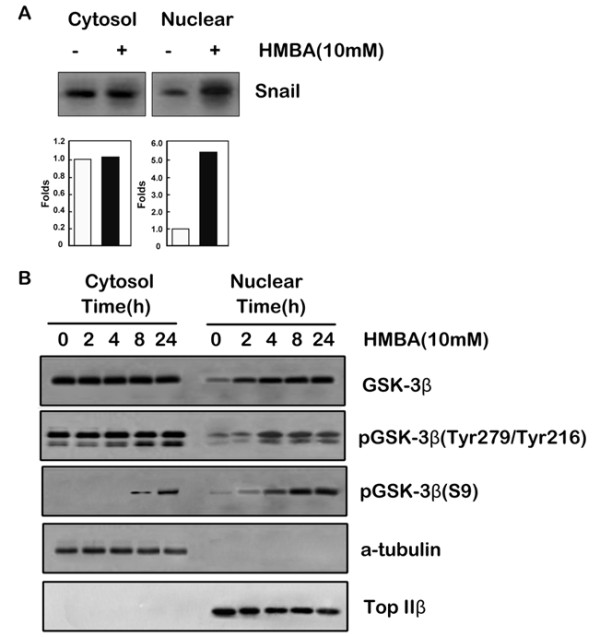
**Treatment with HMBA increased the expression and activity of GSK-3β in the nucleus**. **A**, SGC7901 cells were treated with (+) or without (-) 10 mMHMBA for 24 h, and harvested at the end of the treatment. Cytosol and nuclear fractions were prepared and GSK-3β activity was assayed by in vitro kinase assay using Snail protein as substrate. Phosphorylated Snail protein signals were densitometrically quantitated and expressed as fold-change with respect to untreated control groups. **B**, SGC7901 cells were treated with 10 mM HMBA for various times. Cytosolic and nuclear protein fractions were extracted and western blotting was performed using antibodies to GSK-3β, phospho-GSK-3β (Ser9), phospho-GSK-3α/β (Tyr278/Tyr216), α-tubulin or Topo IIβ.

To test whether GSK-3β was influenced by HMBA treatment, GSK-3β activity was determined by measuring the phosphorylation of recombinant Snail, a well-characterized substrate of GSK-3β [35,36]. GSK-3β is located in the cytosolic and nuclear compartments of cells, but predominantly in the cytoplasm during the G1 phase. Therefore, nuclear and cytoplasmic proteins were fractionated from control and HMBA-treated cells and examined for GSK-3β activity. HMBA treatment resulted in an increase in the activity of nuclear GSK-3β (Figure 3a), and GSK-3β inhibition attenuated HMBA-mediated G1 arrest, indicating a role for GSK-3β in HMBA-induced cell cycle arrest. Ser9 phosphorylation of GSK-3β decreases GSK-3β activity, whereas Tyr216 phosphorylation increases GSK-3β activity [37]. To analyze the mechanisms underlying increased GSK-3β activity caused by HMBA treatment, Ser9-phosphorylated and Tyr216-phosphorylated GSK-3β protein expression was determined using western blotting. HMBA treatment increased nuclear expression levels of total GSK-3β and Tyr216-phosphorylated GSK-3β without affecting their expression in the cytosol (Figure 3b). Interestingly, HMBA treatment increased Ser9-phosphorylated GSK-3β protein expression in both the cytosolic and nuclear fractions. Similar results were obtained following treatment with other HPCs, SAHA and EMBA (Additional file 2). In addition, HPC increased the activity of GSK-3β in the nucleus as demonstrated by *in vitro *kinase assays (Additional file 3). These results suggest that HPC increases nuclear GSK-3β activity irrespective of phosphorylation at Ser9.

### Inhibition of GSK-3β overrides HMBA-induced CDK2 inhibition

**Figure 4:**
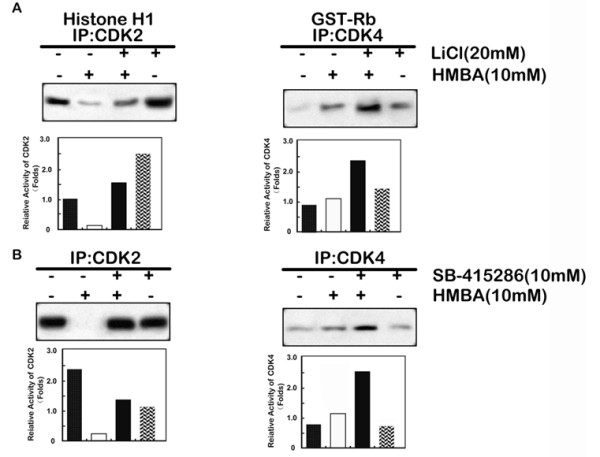
**Inhibition of GSK-3β overrides HMBA-induced CDK2 inhibition**. SGC7901 cells were pre-treated with (+) or without (-) 10 mM LiCl (A) or 10 μM SB-415286 (B) for 30 min followed by combination treatment with 10 mM HMBA for 24 h. Protein extracts were immunoprecipitated with anti-CDK2 or anti-CDK4 antibodies. Resultant immune complexes were analyzed for CDK2 activity using histone H1 as substrate (upper panel) or for CDK4 activity using Rb as substrate (lower panel). Phosphorylated histone H1 or Rb protein signals were quantitated densitometrically and expressed as fold-change with respect to untreated control groups.

Progression through G1 is dependent on CDK2 and CDK4 [38,39]. To determine whether GSK-3β regulation of HMBA-mediated G1 cell cycle arrest occurs via CDK2 or CDK4 inhibition, SGC7901 cells were pre-treated with LiCl (Figure 4a) or SB-415286 (Figure 4b) and then subjected to combination treatment with HMBA for 24 h before immunoprecipitation assays were carried out on the cell lysates using anti-CDK2 or anti-CDK4 antibodies, respectively. In addition, CDK2 or CDK4 activity was determined using an *in vitro *kinase assay. Treatment with HMBA alone inhibited CDK2 activity (top, left panel) but increased CDK4 activity (top, right panel); inhibition of GSK-3β using LiCl or SB-415286 significantly attenuated HMBA inhibition of CDK2 activity (bottom). Treatment with HMBA increased the expression and activity of GSK-3β in the nucleus. Taken together, these results suggest that GSK-3β contributes to HMBA-mediated G1 cell cycle arrest through inhibition of CDK2.

### GSK-3β regulates nuclear p27^Kip1 ^protein expression

**Figure 5:**
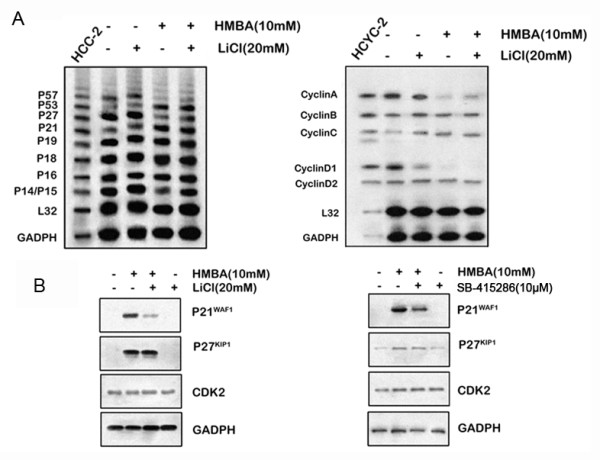
**mRNA expression of genes with regard to cell cycle**. **A**, RNase protection assays were performed using RNA from SGC7901 cells treated with either 10 mM HMBA, 20 mM LiCl, or a combination of HMBA and LiCl for 24 h, hybridized with multi-probes for cell cycle dependent kinase inhibitors (A; hCC-2) or cyclins (B; hCYC-1). **B**, SGC7901 cells were pre-treated with (+) or without (-) 10 mM LiCl (right) or 10 μM SB-415286 (left) for 30 min followed by combination treatment with 10 mM HMBA for 24 h. Whole cell protein extracts were resolved on SDS-PAGE, transferred to PVDF membranes, and immunoblotted with antibodies to p27^Kip1^, p21^Waf1^, CDK2, or β-actin.

To examine the mechanisms underlying HMBA mediated G1 arrest and CDK2 inhibition further, cell cycle-regulatory mRNA expression was analyzed using RPA assays. Treatment with HMBA increased P27^Kip1 ^mRNA expression but decreased p53, p57, P15 (Figure 5A right), cyclin A and cyclin D1 mRNA expression (Figure 5A left). However, treatment with LiCl increased p21^Waf1 ^mRNA expression but did not affect the expression levels of other genes. Similar results were obtained when cells were treated with SB-415286 (Additional file 4). These results suggest that GSK-3β regulation of HMBA-mediated cell cycle arrest does not involve the transcriptional regulation of cell cycle-related genes.

**Figure 6:**
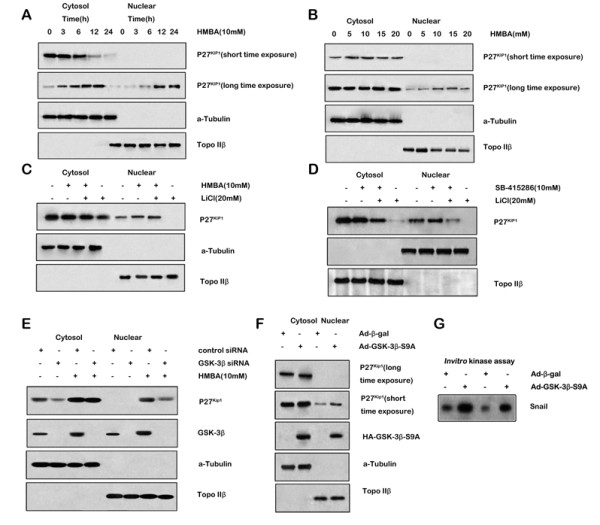
**Nuclear p27^Kip1 ^expression modulated by GSK-3β**. **A& B**, SGC7901 cells were treated with HMBA (10 mM) over a time course (A) or with various concentrations for 24 h. Cytosolic and nuclear protein fractions were extracted and western blotting was performed with antibodies to p27^Kip1^, α-tubulin or Topo IIβ. **C& D**, SGC7901 cells were pre-treated with (+) or without (-) 20 mM LiCl (C) or 10 μM SB-415286 (D) for 30 min followed by combination treatment with 10 mM HMBA for 24 h. Cytosol and nuclear proteins were extracted for analysis of p27^Kip1 ^protein expression. **E**, SGC7901 cells were transfected with siRNA directed to GSK-3β or control siRNA. Twenty-four hours after transfection, cells were treated with HMBA for an additional 24 h. Cytosol and nuclear proteins were extracted for analysis of p27^Kip1 ^protein expression. Knockdown of GSK-3β expression was confirmed by western blotting using anti-GSK-3β antibody. **F**, SGC7901 cells were infected with Ad-HA-GSK-3βS9A or Ad-β-gal at an MOI of 10 pfu/cell. After 48 h incubation, cytosol and nuclear protein were extracted and western blotting performed using anti-p27^Kip1^, anti-HA, and anti-GSK-3 antibodies, respectively using anti-α-tubulin or Topo IIβ as loading control. GSK-3β activities were assayed by in vitro kinase assay using Snail protein as substrate (bottom panel). p27^Kip1 ^signals were quantitated densitometrically and expressed as fold-change with respect to α-tubulin or TopIIβ.

To analyze the mechanisms underlying GSK-3β-associated cell cycle arrest further, the expression of p21^Waf1 ^and p27^Kip1 ^proteins in SGC7901 cells treated with HMBA in the presence or absence of LiCl or SB-415286 was examined. Addition of LiCl (Figure 5B right) or SB-415286 (Figure 5B left) attenuated the induction of p27^Kip1 ^but not p21^Waf1 ^protein expression, suggesting that p27^Kip1 ^participates in the cell cycle transitions regulated by GSK-3β. When p27^Kip1 ^accumulates in the nucleus it binds to CDK2, inhibiting its activity, and eventually induces cell cycle arrest. Furthermore, HMBA (10 mM) increased p27^Kip1 ^protein expression in the cytosol and nucleus from 0 to 24 h after treatment (Figure 6a). HMBA (0-5 mM) increased p27^Kip1 ^expression after 24 h in cytosolic fractions and after 48 h HMBA (5-10 mM) increased p27^Kip1 ^expression in nuclear fractions (Figure 6b). Addition of LiCl (Figure 6c) or SB-415286 (Figure 6d) blocked HMBA-increased p27^Kip1 ^nuclear expression without affecting p27^Kip1 ^expression in the cytosol, suggesting specific regulation of nuclear p27^Kip1 ^expression by GSK-3β. To demonstrate the role of GSK-3β in the regulation of nuclear p27^Kip1 ^expression further, cells were transfected with siRNA directed against GSK-3β (Figure 6e). RNAi-mediated suppression of GSK-3β was confirmed by immunoblotting and attenuated nuclear p27^Kip1 ^induction by HMBA without affecting cytosolic p27^Kip1 ^induction. To confirm the role of GSK-3β in the regulation of nuclear p27^Kip1 ^expression, SGC7901 cells were transfected with a vector encoding the activated form of GSK-3β (GSK-3β-CA) or an empty control vector. Cytosol and nuclear proteins were extracted and western blotting was performed to determine p27^Kip1 ^expression. Transfection of SGC7901 cells with the GSK-3βCA plasmid resulted in increased p27^Kip1 ^in the nuclear fraction (Figure 6F) without affecting p27^Kip1 ^levels in the cytosol. Over-expression of the active form of GSK-3β was confirmed using western blotting and *in vitro *kinase assays using Snail protein as the substrate (Figure 6G). Taken together, these results indicate that GSK-3β participates in the regulation of the cell cycle through the specific regulation of nuclear p27^Kip1 ^protein expression.

### GSK-3β regulates p27^Kip1 ^binding to CDK2

**Figure 7:**
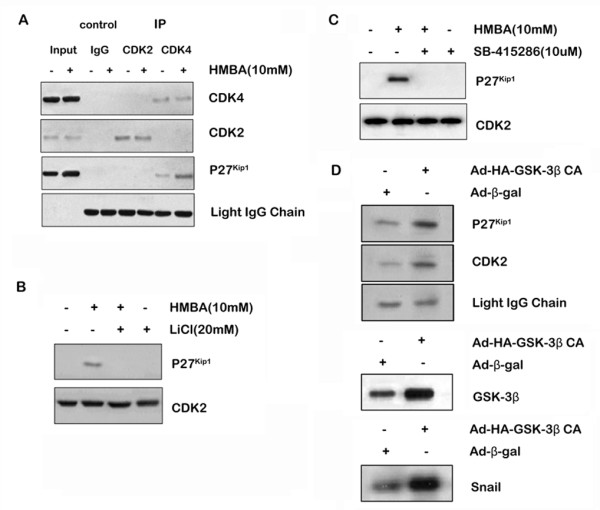
**GSK-3β regulation of p27^Kip^1 association with CDK2**. A, SGC7901 cells were treated with (+) or without (-) 10 mM HMBA for 24 h. Protein extracts were immunoprecipitated with anti-CDK2 or anti-CDK4 antibodies. Normal rabbit IgG was used as the control. The CDK2-or CDK4-associated p27^Kip^1 in the resultant immune complexes was analyzed by western blotting using anti-p27^Kip1 ^antibody with anti-CDK2 or -CDK4 antibody as loading controls. **B&C**, SGC7901 cells were pre-treated with (+) or without (-) 10 mM LiCl (B) or 10 μM SB-415286 (C) for 30 min followed by combination treatment with 10 mM HMBA for 24 h. Protein extracts were immunoprecipitated with an anti-CDK2 antibody. The CDK2 associated p27^Kip^1 in the resultant immune complexes was analyzed similar to A. D, SGC7901 cells were infected with Ad-HA-GSK-3βCA or vector control (Ad-β-gal) at an MOI of 10 pfu/cell. After 48 h incubation, whole cell protein was extracted and immunoprecipitated with anti-CDK2 antibody (upper panel). p27^Kip1 ^was analyzed by western blotting similar to A. Overexpression of HA-tagged GSK-3CA was confirmed by western blotting using anti-GSK-3β antibody (lower panel). GSK-3β activity was assayed by an *in vitro *kinase assay using Snail protein as substrate (bottom panel).

HMBA increased p27^Kip1 ^protein expression, inhibited CDK2 activity and increased CDK4 activity (Figure 4). Extracts from control or HMBA-treated cells were immunoprecipitated to investigate p27^Kip1 ^binding to CDK2 and CDK4. As demonstrated in Figure 7a, HMBA treatment increased the level of p27^Kip1 ^in the complexes immunoprecipitated with anti-CDK2 but not in complexes immunoprecipitated with anti-CDK4. Re-probing the filters with anti-CDK2 and anti-CDK4 antibodies confirmed that the immunoprecipitates from control and HMBA-treated cells contained the same levels of CDK2 and CDK4. Therefore, HMBA appears to cause a selective increase in p27^Kip1 ^binding to CDK2. To analyze whether inhibition of GSK-3β affects the association of p27^Kip1 ^with CDK2, SGC7901 cells were pre-treated with LiCl or SB-415286 and subjected to combination treatment with HMBA for 24 h; whole-cell extracts were immunoprecipitated. Treatment with GSK-3β inhibitors, LiCl (Figure 7b) or SB-415286 (Figure 7c) blocked p27^Kip1 ^binding to CDK2. These results suggest that GSK-3β is essential for HMBA-induced increased p27^Kip1 ^binding to CDK2. To confirm the role of GSK-3β in the regulation of p27^Kip1 ^association with CDK2, SGC7901 cells were transfected with a vector encoding the activated form of GSK-3β or Ad-β-gal. Whole-cell protein was extracted and immunoprecipitated. As presented in Figure 7d, transfection of SGC7901 cells with the GSK-3β-CA vector resulted in an increased level of p27^Kip1 ^in the complexes immunoprecipitated with anti-CDK2 compared with transfection of the control plasmid. This suggests that GSK-3β is not only necessary for HMBA-mediated p27^Kip1 ^binding to CDK2, but also sufficient to increase the association of p27^Kip1 ^with CDK2 in SGC7901 cells.

## Discussion

The PI3-kinase/Akt/GSK-3β signaling pathway has been implicated in the regulation of cell growth, apoptosis and differentiation of various cell types [46]. In the present study, inhibition of GSK-3β using complementary approaches (i.e. chemical inhibition and constitutively active Akt over-expression) attenuated proton pump expression, a measure of gastric-like differentiation, in the gastric tumor-derived SGC7901 cell line.

Cell proliferation and differentiation are traditionally perceived as reciprocal processes, with cell-cycle withdrawal being required for terminal differentiation [40], and P27^Kip1 ^playing an important role [41,42]. Genetic deletion of p27 but not p21 has been shown to affect gastric cell differentiation, whereas forced p27^Kip1 ^expression leads to differentiation, suggesting that p27^Kip1 ^is more important than p21^Waf1 ^in the regulation of gastric cell differentiation. In agreement with these findings, inhibition of GSK-3β attenuated HMBA-mediated gastric cell differentiation and inhibition of GSK-3β blocked HMBA-mediated nuclear p27^Kip1 ^expression, while over-expression of the active form of GSK-3β increased nuclear p27^Kip1 ^expression, suggesting an important role in the regulation of gastric cell differentiation through the regulation of nuclear p27^Kip1 ^expression. Previous studies have demonstrated that inhibition of the PI3-kinase pathway or over-expression of PTEN increases p27^Kip1 ^levels and enhances cell differentiation [43,44]. Consistent with the results presented herein, inactivation of GSK-3β using LiCl results in down-regulation of p27^Kip1 ^and cell cycle progression in primary human T cells [45].

HPC decreases the phosphorylation of Akt [8,47], and therefore decreases the phosphorylation of GSK-3β at Ser9. Ser9 phosphorylation of GSK-3β decreases GSK-3β activity, whereas Tyr216 phosphorylation increases GSK-3β activity [37]. In the present study, HPC decreased Akt phosphorylation significantly in the cytosol but only a minor effect was noted in the nucleus. These results suggest that the induction of GSK-3β activity in the nucleus may not be due to dephosphorylated Akt, which should result in the dephosphorylation of GSK-3β at Ser9, as treating SGC7901 cells with HPC increased GSK-3β phosphorylation at Ser9. HPC increased total GSK-3β protein expression and GSK-3β phosphorylation at Tyr216, but the mechanisms underlying GSK-3β activation in the nucleus by HPC, which could involve novel pathways or additional regulatory elements for GSK-3β, remain to be elucidated.

Nuclear GSK-3β expression is related to cell cycle progression [48]. GSK-3β is predominantly cytoplasmic during the G1 phase [48], but a significant fraction enters the nucleus during the S phase [49,50]. HMBA increased expression and activity of nuclear GSK-3β and induced G1 cell cycle arrest, which was attenuated by GSK-3β inhibition. These results demonstrate a role for GSK-3β in the modulation of G1 cell cycle progression. In addition to attenuation of G1 arrest, treatment with GSK-3β inhibitors increased the cell population in the G2/M phase. GSK-3β may regulate G2/M through the regulation of Chk1 phosphorylation, an important regulator of the G2/M checkpoint [51,52], as SB-415286 and LiCl enhance Chk1 phosphorylation and G2/M arrest by etoposide. Fluxes in levels of GSK-3β in the nucleus at critical periods could be related to the well-documented capacity of nuclear GSK-3β to activate NF-κB [53,54]. Inhibition of NF-κB increases the percentage of cancer cells in the G2/M phase [55]. Nuclear GSK-3β phosphorylates cyclin D1, resulting in the export of cyclin D1 from the nucleus [56,57]. Cyclin D1 plays distinct roles in cell cycle progression through the G1 phase [58]. Collectively, these results reveal an important role for GSK-3β in the regulation of p27^Kip1 ^nuclear localization and CDK2 inhibitory functions on G1/S progression.

The GSK-3β-dependent ubiquitination pathway could serve as a post-transcriptional mechanism regulating the stability of p27^Kip1 ^and its binding to CDK2 in the nucleus. GSK-3β phosphorylates p27^Kip1 ^at S160 and S161, resulting in increased p27^Kip1 ^stability [59]. Therefore, HMBA treatment could result in the phosphorylation of p27^Kip1 ^at S160 and S161 in the nucleus through GSK-3β activation, leading to nuclear p27^Kip1 ^accumulation and increased p27^Kip1 ^binding to CKD2. In conclusion, the present study supports a contributory role of the PI3-kinase/Akt/GSK-3β pathway in the differentiation process of gastric cells. Importantly, the data demonstrated that nuclear GSK-3β increased nuclear p27^Kip1 ^accumulation and p27^Kip1 ^binding to CDK2. Inhibition of CDK2 contributed to HMBA-mediated G1 cell cycle arrest and subsequently to HMBA-mediated gastric cell differentiation.

## Conclusions

The present study identified a novel mechanism whereby GSK-3β affects nuclear p27^Kip1 ^proteolysis, and showed that it participates in the regulation of cell cycle progression and cell differentiation in gastric cells induced by HMBA treatment.

## Competing interests

The authors declare that they have no competing interests.

## Authors' contributions

QLG designed the study, applied for funding and drafted the manuscript. BYL participated in study design and manuscript preparation, and managed the study. ZWW, ZYY and QZ carried out experiments and ensured protocol integrity and collected data. MW and HLY designed the study, undertook statistical analysis and assisted with drafting of the manuscript. LPS participated in the sequence alignment. YYY and ZGZ conceived the study, participated in its design and coordination, and helped to draft the manuscript. All authors read and approved the final manuscript.

## Pre-publication history

The pre-publication history for this paper can be accessed here:

http://www.biomedcentral.com/1471-2407/11/109/prepub

## Supplementary Material

Additional file 1 **Inhibition of GSK-3β by SB-415286 attenuates HMBA-induced cell cycle arrest in SGC7901 cell**. SGC7901 cells were pre-treated with or without 10 μM SB-415286 for 30 min and subjected to combination treatment with 10 mM HMBA for 24 h before quantification of DNA content were carried out using flow cytometry. Click here for file

Additional file 2 **Treatment with EMBA and SAHA activates GSK-3β in the nucleus**. SGC7901 cells were treated with 10 mM EMBA (A) and 10 μM SAHA (B) for various times. Cytosolic and nuclear protein fractions were extracted and western blotting was performed using antibodies to GSK-3β, phospho-GSK-3β (Ser9), phospho-GSK-3α/β (Tyr278/Tyr216), α-tubulin or Topo IIβ. Click here for file

Additional file 3 **EMBA or SAHA treatment activates GSK-3β in the nucleus**. SGC7901 cells were treated with (+) or without (-) 10 mM EMBA (A) and 10 μM SAHA (B) for 24 h, and harvested at the end of the treatment. Cytosolic and nuclear fractions were prepared and GSK-3β activity was assayed by in vitro kinase assay using Snail protein as a substrate of GSK-3β. Click here for file

Additional file 4 **Determination of cell cycle mRNA expression in SGC7901 cells treated with EMBA or SAHA**. RNase protection assays were performed using RNA from SGC7901 cells treated with 10 mM EMBA(A) or 10 μM SAHA(B), 20 mM LiCl, and combination of EMBA(A) or 10 μM SAHA(B) and LiCl for 24 h, hybridized with multi-probes for cell cycle dependent kinase inhibitors (A; hCC-2) or cyclins (B; hCYC-1). Click here for file

## References

[B1] Gupta PB Onder TT Jiang G Tao K Kuperwasser C Weinberg RA Lander ES Identification of selective inhibitors of cancer stem cells by high-throughput screening Cell 2009 138 4 645 659 10.1016/j.cell.2009.06.034 19682730 PMC4892125

[B2] Bodalina UM Hammond KD Gilbert DA Temporal variation in the expression of the p53 protein in proliferating and differentiating murine erythroleukaemia cells Mol Cell Biochem 2007 294 1-2 155 162 10.1007/s11010-006-9255-y 16896537

[B3] Ickowicz Schwartz D Gozlan Y Greenbaum L Babushkina T Katcoff DJ Malik Z Differentiation-dependent photodynamic therapy regulated by porphobilinogen deaminase in B16 melanoma Br J Cancer 2004 90 9 1833 1841 15150593 10.1038/sj.bjc.6601760 PMC2409749

[B4] Yik JH Chen R Nishimura R Jennings JL Link AJ Zhou Q Inhibition of P-TEFb (CDK9/Cyclin T) kinase and RNA polymerase II transcription by the coordinated actions of HEXIM1 and 7SK snRNA Mol Cell 2003 12 4 971 982 10.1016/S1097-2765(03)00388-5 14580347

[B5] Jing Y Xia L Lu M Waxman S The cleavage product deltaPML-RARalpha contributes to all-trans retinoic acid-mediated differentiation in acute promyelocytic leukemia cells Oncogene 2003 22 26 4083 4091 10.1038/sj.onc.1206568 12821942

[B6] Marks PA Richon VM Kiyokawa H Rifkind RA Inducing differentiation of transformed cells with hybrid polar compounds: a cell cycle-dependent process Proc Natl Acad Sci USA 1994 91 22 10251 10254 10.1073/pnas.91.22.10251 7937935 PMC44997

[B7] Dey A Chao SH Lane DP HEXIM1 and the control of transcription elongation: from cancer and inflammation to AIDS and cardiac hypertrophy Cell Cycle 2007 6 15 1856 1863 10.4161/cc.6.15.4556 17671421

[B8] Dey A Wong E Kua N Teo HL Tergaonkar V Lane D Hexamethylene bisacetamide (HMBA) simultaneously targets AKT and MAPK pathway and represses NF kappaB activity: implications for cancer therapy Cell Cycle 2008 7 23 3759 3767 10.4161/cc.7.23.7213 19029824

[B9] Matushansky I Radparvar F Skoultchi AI Manipulating the onset of cell cycle withdrawal in differentiated erythroid cells with cyclin-dependent kinases and inhibitors Blood 2000 96 8 2755 2764 11023509

[B10] Karam SM Lineage commitment and maturation of epithelial cells in the gut Front Biosci 1999 4 D286 298 10.2741/Karam 10077541

[B11] Brittan M Wright NA Gastrointestinal stem cells J Pathol 2002 197 4 492 509 10.1002/path.1155 12115865

[B12] Mishra L Banker T Murray J Byers S Thenappan A He AR Shetty K Johnson L Reddy EP Liver stem cells and hepatocellular carcinoma Hepatology 2009 49 1 318 329 10.1002/hep.22704 19111019 PMC2726720

[B13] Nitta T Egashira Y Akutagawa H Edagawa G Kurisu Y Nomura E Tanigawa N Shibayama Y Study of clinicopathological factors associated with the occurrence of synchronous multiple gastric carcinomas Gastric Cancer 2009 12 1 23 30 10.1007/s10120-008-0493-4 19390928

[B14] Li P Li C Zhao X Zhang X Nicosia SV Bai W p27(Kip1) stabilization and G(1) arrest by 1,25-dihydroxyvitamin D(3) in ovarian cancer cells mediated through down-regulation of cyclin E/cyclin-dependent kinase 2 and Skp1-Cullin-F-box protein/Skp2 ubiquitin ligase J Biol Chem 2004 279 24 25260 25267 10.1074/jbc.M311052200 15075339

[B15] Baldassarre G Barone MV Belletti B Sandomenico C Bruni P Spiezia S Boccia A Vento MT Romano A Pepe S Key role of the cyclin-dependent kinase inhibitor p27kip1 for embryonal carcinoma cell survival and differentiation Oncogene 1999 18 46 6241 6251 10.1038/sj.onc.1203031 10597222

[B16] Glick RD Swendeman SL Coffey DC Rifkind RA Marks PA Richon VM La Quaglia MP Hybrid polar histone deacetylase inhibitor induces apoptosis and CD95/CD95 ligand expression in human neuroblastoma Cancer Res 1999 59 17 4392 4399 10485488

[B17] Stambolic V Woodgett JR Mitogen inactivation of glycogen synthase kinase-3 beta in intact cells via serine 9 phosphorylation Biochem J 1994 303 Pt 3 701 704 7980435 10.1042/bj3030701 PMC1137602

[B18] Harwood AJ Plyte SE Woodgett J Strutt H Kay RR Glycogen synthase kinase 3 regulates cell fate in Dictyostelium Cell 1995 80 1 139 148 10.1016/0092-8674(95)90458-1 7813009

[B19] Gattinoni L Zhong XS Palmer DC Ji Y Hinrichs CS Yu Z Wrzesinski C Boni A Cassard L Garvin LM Wnt signaling arrests effector T cell differentiation and generates CD8+ memory stem cells Nat Med 2009 15 7 808 813 10.1038/nm.1982 19525962 PMC2707501

[B20] Muller MR Sasaki Y Stevanovic I Lamperti ED Ghosh S Sharma S Gelinas C Rossi DJ Pipkin ME Rajewsky K Requirement for balanced Ca/NFAT signaling in hematopoietic and embryonic development Proc Natl Acad Sci USA 2009 106 17 7034 7039 10.1073/pnas.0813296106 19351896 PMC2678457

[B21] Ougolkov AV Fernandez-Zapico ME Bilim VN Smyrk TC Chari ST Billadeau DD Aberrant nuclear accumulation of glycogen synthase kinase-3beta in human pancreatic cancer: association with kinase activity and tumor dedifferentiation Clin Cancer Res 2006 12 17 5074 5081 10.1158/1078-0432.CCR-06-0196 16951223 PMC2692690

[B22] Yook JI Li XY Ota I Hu C Kim HS Kim NH Cha SY Ryu JK Choi YJ Kim J A Wnt-Axin2-GSK3beta cascade regulates Snail1 activity in breast cancer cells Nat Cell Biol 2006 8 12 1398 1406 10.1038/ncb1508 17072303

[B23] Marchetti A Colletti M Cozzolino AM Steindler C Lunadei M Mancone C Tripodi M ERK5/MAPK is activated by TGFbeta in hepatocytes and required for the GSK-3beta-mediated Snail protein stabilization Cell Signal 2008 20 11 2113 2118 10.1016/j.cellsig.2008.08.002 18760348

[B24] Wei M Liu B Su L Li J Zhang J Yu Y Yan M Yang Z Chen X Liu J A novel plant homeodomain finger 10-mediated antiapoptotic mechanism involving repression of caspase-3 in gastric cancer cells Mol Cancer Ther 9 6 1764 1774 10.1158/1535-7163.MCT-09-1162 20530714

[B25] Wang YW Qu Y Li JF Chen XH Liu BY Gu QL Zhu ZG In vitro and in vivo evidence of metallopanstimulin-1 in gastric cancer progression and tumorigenicity Clin Cancer Res 2006 12 16 4965 4973 10.1158/1078-0432.CCR-05-2316 16914586

[B26] Ding QM Ko TC Evers BM Caco-2 intestinal cell differentiation is associated with G1 arrest and suppression of CDK2 and CDK4 Am J Physiol 1998 275 5 Pt 1 C1193 1200 9814966 10.1152/ajpcell.1998.275.5.C1193

[B27] Watcharasit P Bijur GN Zmijewski JW Song L Zmijewska A Chen X Johnson GV Jope RS Direct, activating interaction between glycogen synthase kinase-3beta and p53 after DNA damage Proc Natl Acad Sci USA 2002 99 12 7951 7955 10.1073/pnas.122062299 12048243 PMC123001

[B28] Boyle WJ Smeal T Defize LH Angel P Woodgett JR Karin M Hunter T Activation of protein kinase C decreases phosphorylation of c-Jun at sites that negatively regulate its DNA-binding activity Cell 1991 64 3 573 584 10.1016/0092-8674(91)90241-P 1846781

[B29] Zhang G Wang G Wang S Li Q Ouyang G Peng X Applying proteomic methodologies to analyze the effect of hexamethylene bisacetamide (HMBA) on proliferation and differentiation of human gastric carcinoma BGC-823 cells The International Journal of Biochemistry & Cell Biology 2004 36 8 1613 1623 10.1016/j.biocel.2004.01.021 15147739

[B30] Wang Q Zhou Y Wang X Evers BM p27 Kip1 nuclear localization and cyclin-dependent kinase inhibitory activity are regulated by glycogen synthase kinase-3 in human colon cancer cells Cell Death Differ 2008 15 5 908 919 10.1038/cdd.2008.2 18408738 PMC2432084

[B31] Johnson M Sharma M Jamieson C Henderson JM Mok MT Bendall L Henderson BR Regulation of beta-catenin trafficking to the membrane in living cells Cell Signal 2009 21 2 339 348 10.1016/j.cellsig.2008.11.004 19036347

[B32] MacAulay K Blair AS Hajduch E Terashima T Baba O Sutherland C Hundal HS Constitutive activation of GSK3 down-regulates glycogen synthase abundance and glycogen deposition in rat skeletal muscle cells J Biol Chem 2005 280 10 9509 9518 10.1074/jbc.M411648200 15632169

[B33] Zhang JY Tao LY Liang YJ Yan YY Dai CL Xia XK She ZG Lin YC Fu LW Secalonic acid D induced leukemia cell apoptosis and cell cycle arrest of G(1) with involvement of GSK-3beta/beta-catenin/c-Myc pathway Cell Cycle 2009 8 15 2444 2450 10.4161/cc.8.15.9170 19571678

[B34] Jiang H Guo W Liang X Rao Y Both the establishment and the maintenance of neuronal polarity require active mechanisms: critical roles of GSK-3beta and its upstream regulators Cell 2005 120 1 123 135 15652487 10.1016/j.cell.2004.12.033

[B35] Schlessinger K Hall A GSK-3beta sets Snail's pace Nat Cell Biol 2004 6 10 913 915 10.1038/ncb1004-913 15459715

[B36] Zhou BP Deng J Xia W Xu J Li YM Gunduz M Hung MC Dual regulation of Snail by GSK-3beta-mediated phosphorylation in control of epithelial-mesenchymal transition Nat Cell Biol 2004 6 10 931 940 10.1038/ncb1173 15448698

[B37] Liang MH Chuang DM Regulation and function of glycogen synthase kinase-3 isoforms in neuronal survival J Biol Chem 2007 282 6 3904 3917 10.1074/jbc.M605178200 17148450

[B38] Gladden AB Diehl JA Cell cycle progression without cyclin E/CDK2: breaking down the walls of dogma Cancer Cell 2003 4 3 160 162 10.1016/S1535-6108(03)00217-4 14522248

[B39] King RW Deshaies RJ Peters JM Kirschner MW How proteolysis drives the cell cycle Science 1996 274 5293 1652 1659 10.1126/science.274.5293.1652 8939846

[B40] Lin CH Jackson AL Guo J Linsley PS Eisenman RN Myc-regulated microRNAs attenuate embryonic stem cell differentiation EMBO J 2009 19745813 10.1038/emboj.2009.254 PMC2744176

[B41] Bossenmeyer-Pourie C Kannan R Ribieras S Wendling C Stoll I Thim L Tomasetto C Rio MC The trefoil factor 1 participates in gastrointestinal cell differentiation by delaying G1-S phase transition and reducing apoptosis J Cell Biol 2002 157 5 761 770 10.1083/jcb200108056 12034770 PMC2173421

[B42] Wiksten JP Lundin J Nordling S Kokkola A von Boguslawski K Haglund C The prognostic value of p27 in gastric cancer Oncology 2002 63 2 180 184 10.1159/000063813 12239454

[B43] Ma XM Liu Y Guo JW Liu JH Zuo LF Relation of overexpression of S phase kinase-associated protein 2 with reduced expression of p27 and PTEN in human gastric carcinoma World J Gastroenterol 2005 11 42 6716 6721 16425372 10.3748/wjg.v11.i42.6716 PMC4355772

[B44] Lee JY Kang MB Jang SH Qian T Kim HJ Kim CH Kim Y Kong G Id-1 activates Akt-mediated Wnt signaling and p27(Kip1) phosphorylation through PTEN inhibition Oncogene 2009 28 6 824 831 10.1038/onc.2008.451 19079342

[B45] Henson SM Franzese O Macaulay R Libri V Azevedo RI Kiani-Alikhan S Plunkett FJ Masters JE Jackson S Griffiths SJ KLRG1 signaling induces defective Akt (ser473) phosphorylation and proliferative dysfunction of highly differentiated CD8+ T cells Blood 2009 113 26 6619 6628 10.1182/blood-2009-01-199588 19406987

[B46] Qiao M Sheng S Pardee AB Metastasis and AKT activation Cell Cycle 2008 7 19 2991 2996 10.4161/cc.7.19.6784 18818526

[B47] Contreras X Barboric M Lenasi T Peterlin BM HMBA releases P-TEFb from HEXIM1 and 7SK snRNA via PI3K/Akt and activates HIV transcription PLoS Pathog 2007 3 10 1459 1469 10.1371/journal.ppat.0030146 17937499 PMC2014796

[B48] Takahashi-Yanaga F Sasaguri T GSK-3beta regulates cyclin D1 expression: a new target for chemotherapy Cell Signal 2008 20 4 581 589 10.1016/j.cellsig.2007.10.018 18023328

[B49] Meares GP Jope RS Resolution of the nuclear localization mechanism of glycogen synthase kinase-3: functional effects in apoptosis J Biol Chem 2007 282 23 16989 17001 10.1074/jbc.M700610200 17438332 PMC1948884

[B50] Wojcik EJ A mitotic role for GSK-3beta kinase in Drosophila Cell Cycle 2008 7 23 3699 3708 10.4161/cc.7.23.7179 19029800 PMC6713227

[B51] Melixetian M Klein DK Sorensen CS Helin K NEK11 regulates CDC25A degradation and the IR-induced G2/M checkpoint Nat Cell Biol 2009 19734889 10.1038/ncb1969

[B52] Gao D Inuzuka H Korenjak M Tseng A Wu T Wan L Kirschner M Dyson N Wei W Cdh1 regulates cell cycle through modulating the claspin/Chk1 and the Rb/E2F1 pathways Mol Biol Cell 2009 20 14 3305 3316 10.1091/mbc.E09-01-0092 19477924 PMC2710831

[B53] Wilson W Baldwin AS Maintenance of constitutive IkappaB kinase activity by glycogen synthase kinase-3alpha/beta in pancreatic cancer Cancer Res 2008 68 19 8156 8163 10.1158/0008-5472.CAN-08-1061 18829575 PMC2647811

[B54] Kwon O Kim KA He L Kim SO Kim MS Cha EY Yoon BD Sok DE Jung M Ahn JS Ionizing radiation can induce GSK-3beta phosphorylation and NF-kappaB transcriptional transactivation in ATM-deficient fibroblasts Cell Signal 2008 20 4 602 612 10.1016/j.cellsig.2007.10.022 18243662

[B55] Yan X Shen H Jiang H Zhang C Hu D Wang J Wu X External Qi of Yan Xin Qigong induces G2/M arrest and apoptosis of androgen-independent prostate cancer cells by inhibiting Akt and NF-kappa B pathways Mol Cell Biochem 2008 310 1-2 227 234 10.1007/s11010-007-9684-2 18080802

[B56] Pontano LL Diehl JA DNA damage-dependent cyclin D1 proteolysis: GSK3beta holds the smoking gun Cell Cycle 2009 8 6 824 827 10.4161/cc.8.6.7889 19221502 PMC3703625

[B57] Pontano LL Aggarwal P Barbash O Brown EJ Bassing CH Diehl JA Genotoxic stress-induced cyclin D1 phosphorylation and proteolysis are required for genomic stability Mol Cell Biol 2008 28 23 7245 7258 10.1128/MCB.01085-08 18809569 PMC2593367

[B58] Serrano M Hannon GJ Beach D A new regulatory motif in cell-cycle control causing specific inhibition of cyclin D/CDK4 Nature 1993 366 6456 704 707 10.1038/366704a0 8259215

[B59] Surjit M Lal SK Glycogen synthase kinase-3 phosphorylates and regulates the stability of p27kip1 protein Cell Cycle 2007 6 5 580 588 10.4161/cc.6.5.3899 17351340

