# Supplementary figures and images for: P27Kip1, regulated by glycogen synthase kinase-3β, results in HMBA-induced differentiation of human gastric cancer cells
Source: BMC Cancer. 2011 Mar 27;11:109. doi: 10.1186/1471-2407-11-109 (PMC3078896; doi:10.1186/1471-2407-11-109)

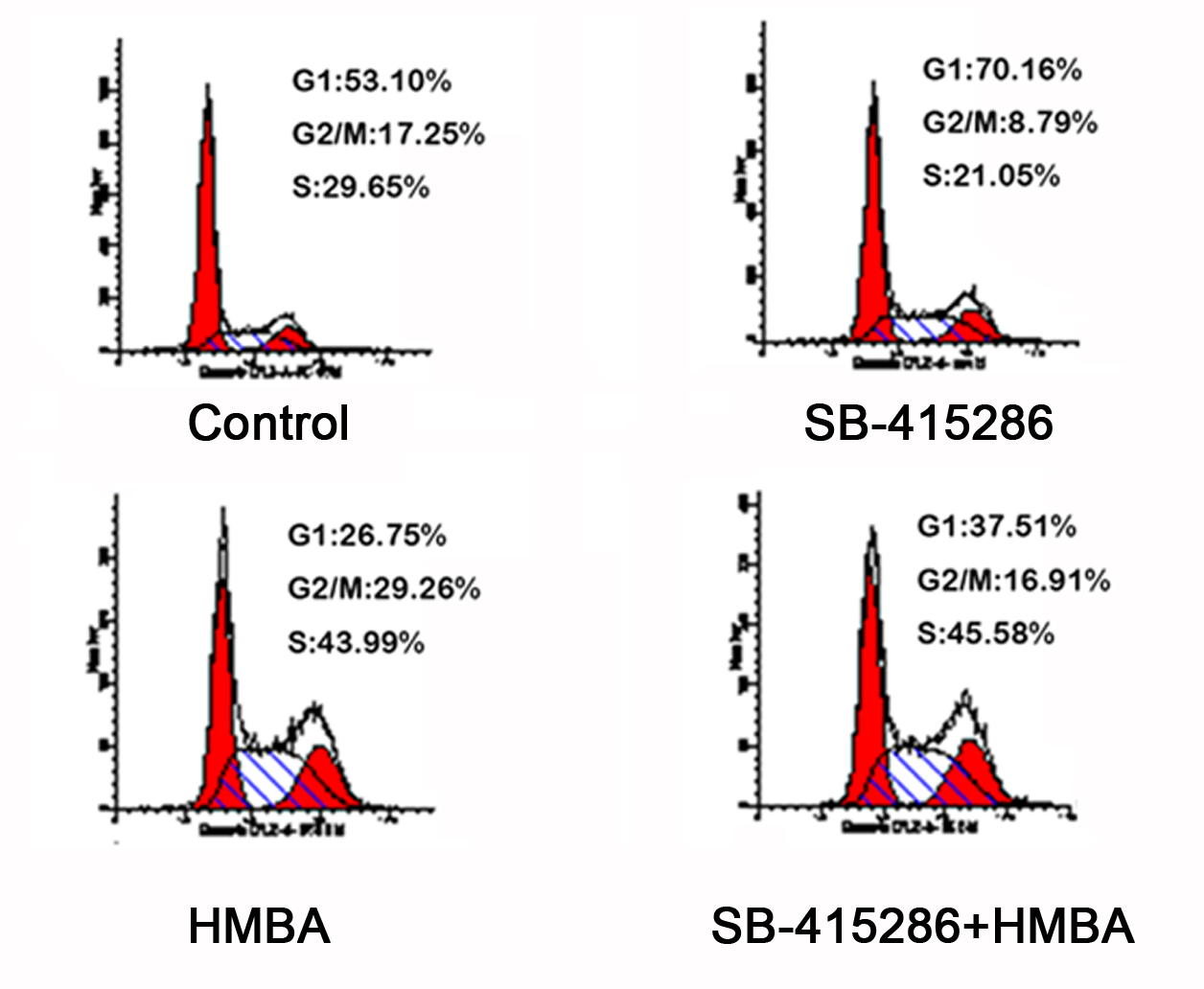

Supplement: Additional file 1 — Inhibition of GSK-3β by SB-415286 attenuates HMBA-induced cell cycle arrest in SGC7901 cell. SGC7901 cells were pre-treated with or without 10 μM SB-415286 for 30 min and subjected to combination treatment with 10 mM HMBA for 24 h before quantification of DNA content were carried out using flow cytometry. [file 1471-2407-11-109-S1.TIFF]

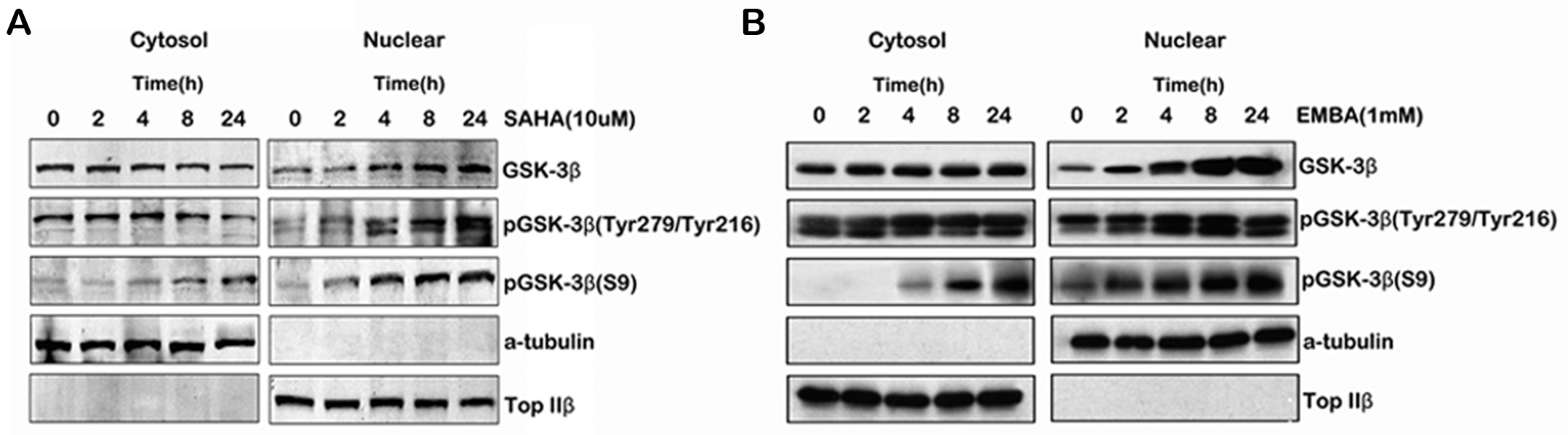

Supplement: Additional file 2 — Treatment with EMBA and SAHA activates GSK-3β in the nucleus. SGC7901 cells were treated with 10 mM EMBA (A) and 10 μM SAHA (B) for various times. Cytosolic and nuclear protein fractions were extracted and western blotting was performed using antibodies to GSK-3β, phospho-GSK-3β (Ser9), phospho-GSK-3α/β (Tyr278/Tyr216), α-tubulin or Topo IIβ. [file 1471-2407-11-109-S2.TIFF]

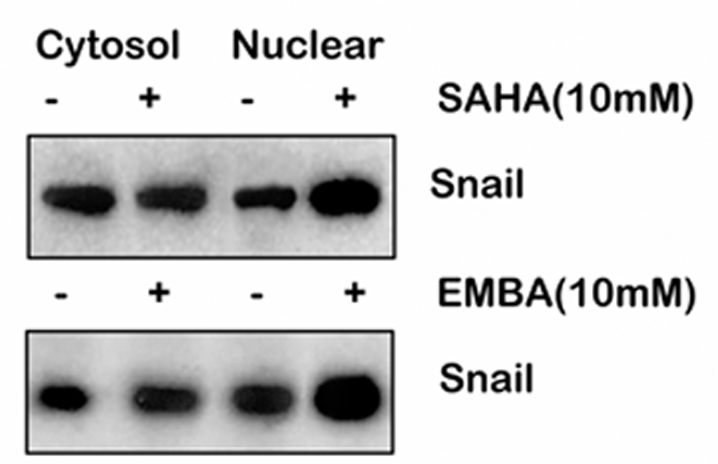

Supplement: Additional file 3 — EMBA or SAHA treatment activates GSK-3β in the nucleus. SGC7901 cells were treated with (+) or without (-) 10 mM EMBA (A) and 10 μM SAHA (B) for 24 h, and harvested at the end of the treatment. Cytosolic and nuclear fractions were prepared and GSK-3β activity was assayed by in vitro kinase assay using Snail protein as a substrate of GSK-3β. [file 1471-2407-11-109-S3.TIFF]

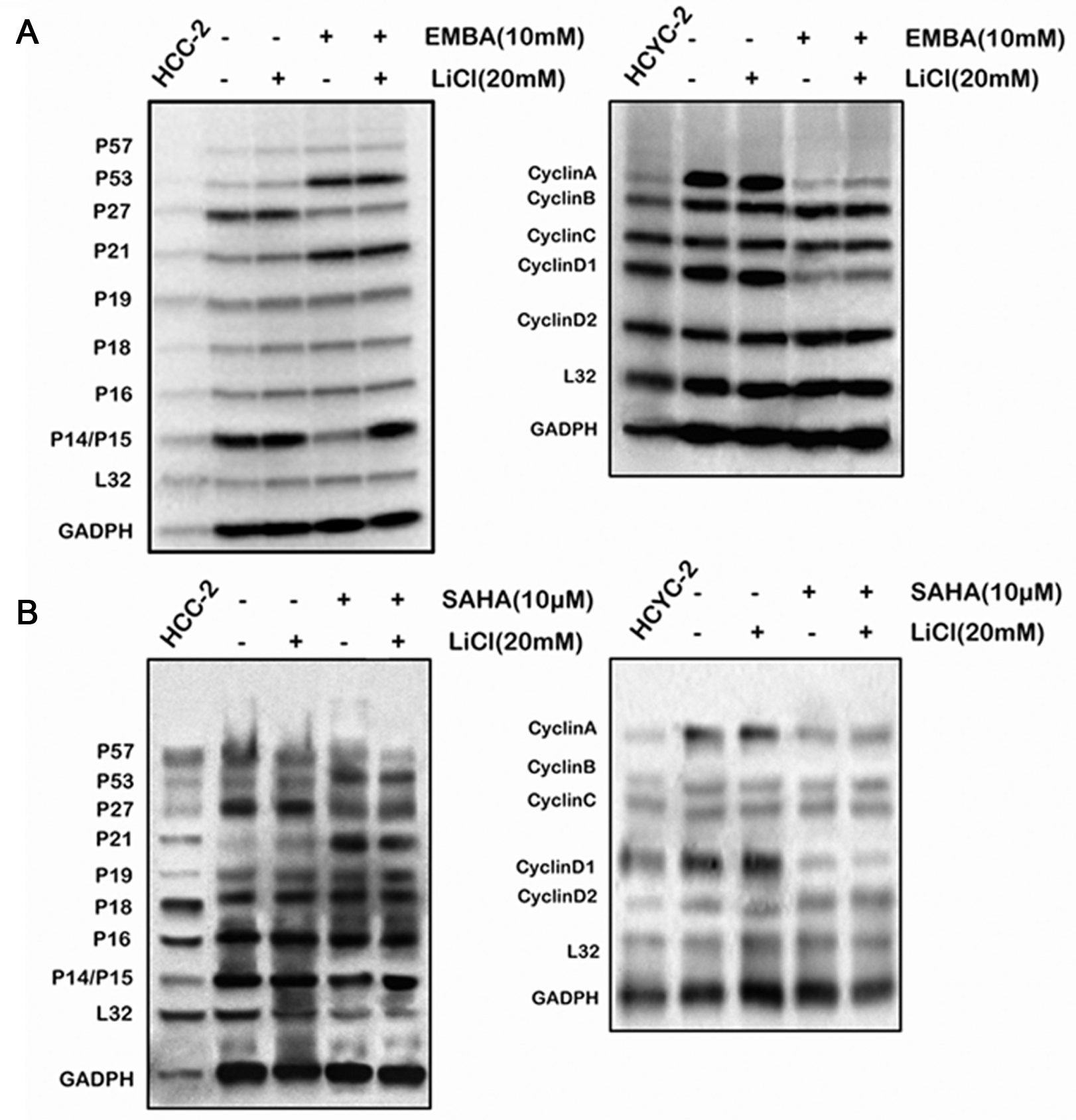

Supplement: Additional file 4 — Determination of cell cycle mRNA expression in SGC7901 cells treated with EMBA or SAHA. RNase protection assays were performed using RNA from SGC7901 cells treated with 10 mM EMBA(A) or 10 μM SAHA(B), 20 mM LiCl, and combination of EMBA(A) or 10 μM SAHA(B) and LiCl for 24 h, hybridized with multi-probes for cell cycle dependent kinase inhibitors (A; hCC-2) or cyclins (B; hCYC-1). [file 1471-2407-11-109-S4.TIFF]
